# Performance improvement of solar still by water mass splitting arrangement

**DOI:** 10.1038/s41598-025-15849-1

**Published:** 2025-08-22

**Authors:** Emmanuel Agbo Tei, Rasool Mohideen, Syed Noman, Muthu Manokar Athikesavan

**Affiliations:** 1 Mechanical Department, School of Engineering, Cape Coast Technical University, P.O. Box DL50, Cape Coast, Ghana; 2 Department of Mechanical Engineering, B.S. Abdur Rahman Crescent Institute of Science and Technology, Vandalur, Chennai, 600 048 India; 3 Department of Electrical and Electronics Engineering, SRM TRP Engineering College, Irungalur, Tiruchirappalli, Tamil Nadu 621105 India

**Keywords:** Solar still, Low-cost energy storage, Black rubber mat, Distilled water, Solar energy, Mechanical engineering

## Abstract

The study explored the use of a black rubber mat as a cost-effective material for energy storage and water splitting, and its potential for enhancing potable water production. In this research, a solar still (SS) and a modified version incorporating a rubber mat were designed and tested. The rubber mat was placed at the bottom of the conventional tubular SS, and the performance of the experimental work was evaluated on 09-10-2019, from 8:00 am to 6:00 pm. The yield of the standard solar still was 1.99 kg, while the modified version with the rubber mat attained a highest water generation of 0.60 kg, with a total output of 2.82 kg. The thermal efficacy was calculated for a modified model, which used a rubber mat, and was 54.80%, significantly higher than the 34.90% of the unmodified version, an improvement of 19.9%. Furthermore, with the addition of a black rubber mat, the upgraded solar’s second law (Exergy) efficiency still reached 2.94%, compared to 1.78% for the standard version, representing a 1.16% increase.

**Supplementary Information:**

The online version contains supplementary material available at 10.1038/s41598-025-15849-1.

## Introduction

Water, a vital natural resource, exists in solid, liquid, or vapour forms^1^. The majority of the ocean constitutes of saltwater and this oceanic water is occupying a land area of 71% of the earth’s surface, Saltwater makes up the majority of the Earth’s water, with oceans covering about 71% of the surface, leaving only a small fraction as accessible freshwater for human use^2,3^. Water can be categorized into three primary sources: surface water, groundwater, and rainwater^4^. Surface water bodies are classified as ponds, rivers, and lakes, while groundwater encompasses wells and springs. Global population growth is projected to lead to a 40% shortage in water supply for drinking and irrigation purposes^5^. Water scarcity has become a significant challenge worldwide. To address this, nations must enhance water management systems, conserve existing resources, recycle wastewater, and develop innovative methods to increase water storage and service capacity^6^.

In arid regions like the Middle East, desalination, which converts seawater into potable water, has become essential^7^. Solar stills (SS) offer a cost-effective and straightforward desalination method, where saline water is evaporated by the sun and condensed into freshwater^8^. This age-old technique is still used in areas where water scarcity or unsuitable groundwater for drinking is a concern. Solar stills are particularly useful in remote locations, away from cities, to support small populations. Solar energy, as a renewable resource, also reduces reliance on coal and gas power plants^9^. The process mimics natural rain production through evaporation and condensation, but it is time-consuming and only produces around six litres of water on a sunny. Although solar stills are effective in certain areas, challenges such as slow production rates and limited raw material availability remain, prompting ongoing research to improve efficiency.

Advances in technology are driving down the cost of installing and producing solar stills (SS), with various modifications enhancing their efficiency. SS designs include Tubular SS, Inclined SS, Single Slope SS, and Double Slope SS, which have been adapted using wicks, rectangular fins, phase change materials, and carbon nanoparticles to increase productivity^10^. With their high solar potential, African nations stand to benefit the most from solar energy applications. However, solar energy isn’t exclusive to regions with abundant sunshine; for instance, Germany generates over a third of its electricity from solar power^8^, despite its limited sunny days. Taghvaei et al.^11^ studied water depth and its impact on SS performance over 10 days, a unique approach compared to shorter studies. They found that deeper water levels improved SS yield and efficiency, even when the water supply was limited.

Additional research supports these findings. Manokar et al.^12^ studied the effects of surface area along with the insulation on SS efficiency, showing that increased surface area and insulation enhance water output. Production rates improved significantly when water depth was optimized. Kabeel et al.^13^ studied a Triangle Shaped Pyramid SS (TSPSS) with an absorber plate coated with TiO_2_ nano-particles, which increased water temperature and yield by 6.1% compared to traditional models. Similarly, Sebaey et al.^14^ introduced a Single Slope Double Basin Solar Still (SSDBSS), which improved daily water production by 59.9%, achieving a thermal efficiency of 61.3%. These studies highlight the importance of design innovations, material enhancements, and water level optimization in boosting the efficiency and productivity of solar stills for desalination purposes. Kabeel et al.^15^ investigated the efficiency of TSS supplemented with a cooling cover. This experimental arrangement comprised a transparent solar tube paired with a water basin of black colour to optimize solar absorption and promote evaporation processes. A range of water depths (0.5, 1, 2, and 3 cm) alongside varying water flow rates (1, 2, 3, and 4 L/h) were systematically evaluated. The findings revealed that the yields obtained without cooling at several depth of water for experimentation of the order of 0.5, 1, 2, and 3 cm were 4.5, 4.2, 3.54, and 3.09 L/m², respectively, whereas the incorporation of cooling elevated the yields to 5.42, 5.85, 5.53, and 5.23 L/m². The optimal yield of 5.85 L/m² was recorded at a cooling flow rate of 2 L/h, while the peak production without cooling, quantified at 4.5 L/m², was noted at a water depth of 0.5 cm. Rajamanickam and Ragupathy^16^ this study examined the influence of water depth on mass transfer and the thermal efficiency of the phenomena occurring within a Sealed Single Basin Double Slope Solar Still (SBDSSS) constructed from galvanized iron. The research conducted a comparative analysis with a Single Slope Solar Still (SSS) utilizing transparent glass and evaluated water depths of 0.01, 0.025, 0.05, and 0.075 m. The results indicated that the SBDSSS attained a maximum daily freshwater production of 3.07 L/m² at a water depth of 0.01 m, in contrast to the SSS, which produced 2.34 L/m², with yield diminishing as water depths increased. Somanchi et al.^17^ conducted a comprehensive investigation into the implications of incorporating phase change materials, such as Magnesium Sulfate Heptahydrate (MgSO_4_·7H_2_O), on the operational efficacy of stainless steel solar still. Integrating this specific material enhanced the efficiency of the water distillation process. Khalifa and Hamood^18^ examined the correlation between the depth of brine and the productivity of freshwater, demonstrating that an increase in the depth of brine led to a reduction in solar still efficiency by approximately 48%, with productivity diminishing as brine depth ascended from 1 to 10 cm. Rahul et al.^19^ explored Inverted Absorber Solar Stills (IASS) versus Single Slope SS. They found that IASS significantly outperformed SSSS at several saltwater depths, especially in the presence of total dissolved solids (TDS). Elango et al.^20^ compared SBDSSS and DBDSSS and discovered that DBDSSS with insulation achieved a maximum daily production of 5.327 L/m² at a 1 cm water level, outperforming SBDSSS, which produced 4.401 L/m². Valsaraj^21^ examined the working conditions of SSBSS using a floating aluminium sheet as an absorber. The study showed that this design increased production by 43% at a 90 mm water depth, with insulation and condensers further enhancing performance. Tiwari and Tiwari^22^ investigated the relationship between internal heat transfer coefficients and water depth in an S inclined at 30°. The study demonstrated that convective and evaporative HTC substantially increased productivity during summer and at lower water depths. Sathyamurthy et al.^23^ investigated the influence of the mass of saline water on the efficiency of a Triangular Pyramid Solar Still (TPSS) incorporating Latent Heat Energy Storage (LHTESS). The study found that reducing the water mass increased potable water yield. TPSS with LHTESS produced 5.5 L/m², 35% higher than TPSS without LHTESS (3.5 L/m²), with higher production observed during nocturnal periods at lower water masses. Kumar et al.^24^ introduced a novel method of combining Inclined Solar Stills (ISS) with TPSS. The integrated system achieved yields of 7.52, 5.9, and 5.66 kg/m² at different depths of saline water in the form of 0.02, 0.04, and 0.06 m for the aforementioned yield values, representing a 79.05% increase over TPSS alone, which yielded 4.2, 3, and 2.16 kg/m², respectively. El-Sebaey et al.^14^ compared the performance of a SSSS and a Single Slope Double Basin Solar Still (SSDBSS). The SSDBSS outperformed the SSSS at all water depths, producing 2.855, 2.445, 2.123, and 1.935 L/m² at 2, 3, 4, and 5 cm depths, respectively, while SSSS yielded 1.785, 1.510, 1.300, and 1.190 L/m². A yield reduction of 14.36% occurred between 2 and 3 cm in SSDBSS, while SSSS saw reductions of 25.64% and 32.24% as water depth increased from 2 to 3 cm and 2 to 5 cm, respectively. Nougriaya et al.^25^ reviewed solar stills with water depths ranging from 1 to 15 cm, finding optimal production between 1 and 2 cm. Factors such as solar radiation, water depth, and nano-particle use affected performance, with paraffin as a PCM improving efficiency. Phadatare and Verma^26^ evaluated water depth’s impact on the transition of mass and heat interior in a Plastic Solar Still with SBSS showing yields of 2.1, 1.9, 1.8, 1.75, 1.8, and 1.85 L/m² at depths of 2, 4, 6, 8, 10, and 12 cm. Maximum productivity occurred at 2 cm, with efficiency ranging from 10 to 34%. Modi et al.^27^ developed a Spherical Basin SS through separate water heating and condensation sections. Daily yields of 3.5409, 4.7860, 6.7174, 7.4744, and 8.2596 L/m² were recorded at water masses of 1, 2, 3, 4, and 5 L, with effectiveness increasing from 19.56 to 39.06%, demonstrating a positive correlation between yield and water mass. Murugavel et al.^28^ conducted a comparative study aimed at improving the freshwater productivity of Double Slope Solar Still (DSSS) by incorporating several wick elements in the form of sponge sheets, several pieces of waste cotton material, light jute material, cotton in black colour and coir mars in addition to integrating aluminium rectangular fins. The study found that black cotton provided the highest yield among wick materials, producing 3.49 kg/day. Furthermore, when 65 mm and 45 mm aluminium fins were wrapped in cotton and jute cloth and arranged lengthwise, the yield increased to a maximum of 3.58 kg/day, surpassing black cotton alone.

### Novelty and objectives

Recent studies have expanded on this approach by testing low-cost or repurposed materials to improve solar still performance^29–33^. Researchers have also examined the use of nanomaterials^34–37^ and various wick materials^38–40^. Hybrid systems^12,41,42^ have also been explored to boost solar still output. Moreover, materials like sodium nitrate (NaNO_3_) and aluminium oxide (Al_2_O_3_)^43^, black glass balls, black granite, white marble stones^44^, quartz rocks, mild steel, red bricks^45^, and paraffin wax^46^ have been employed to increase daily yields. Recently, black cotton wicks and paraffin have also been utilized^47^, different shapes of basin liners^48^, flat and concave basin^49^, Black glass balls^50^, different absorbing materials^51^, rubber^52^. Still, no documented research has explored using black rubber hole mats as a low-cost energy storage material in solar stills. This study introduces a novel approach by investigating black rubber mats for enhancing the operating conditioning of the solar still. Black rubber mat enhanced solar absorptivity, increased surface area via holes, thermal mass and heat retention, and promotes thin-film evaporation.

## Description of experimental work

**Fig. 1:**
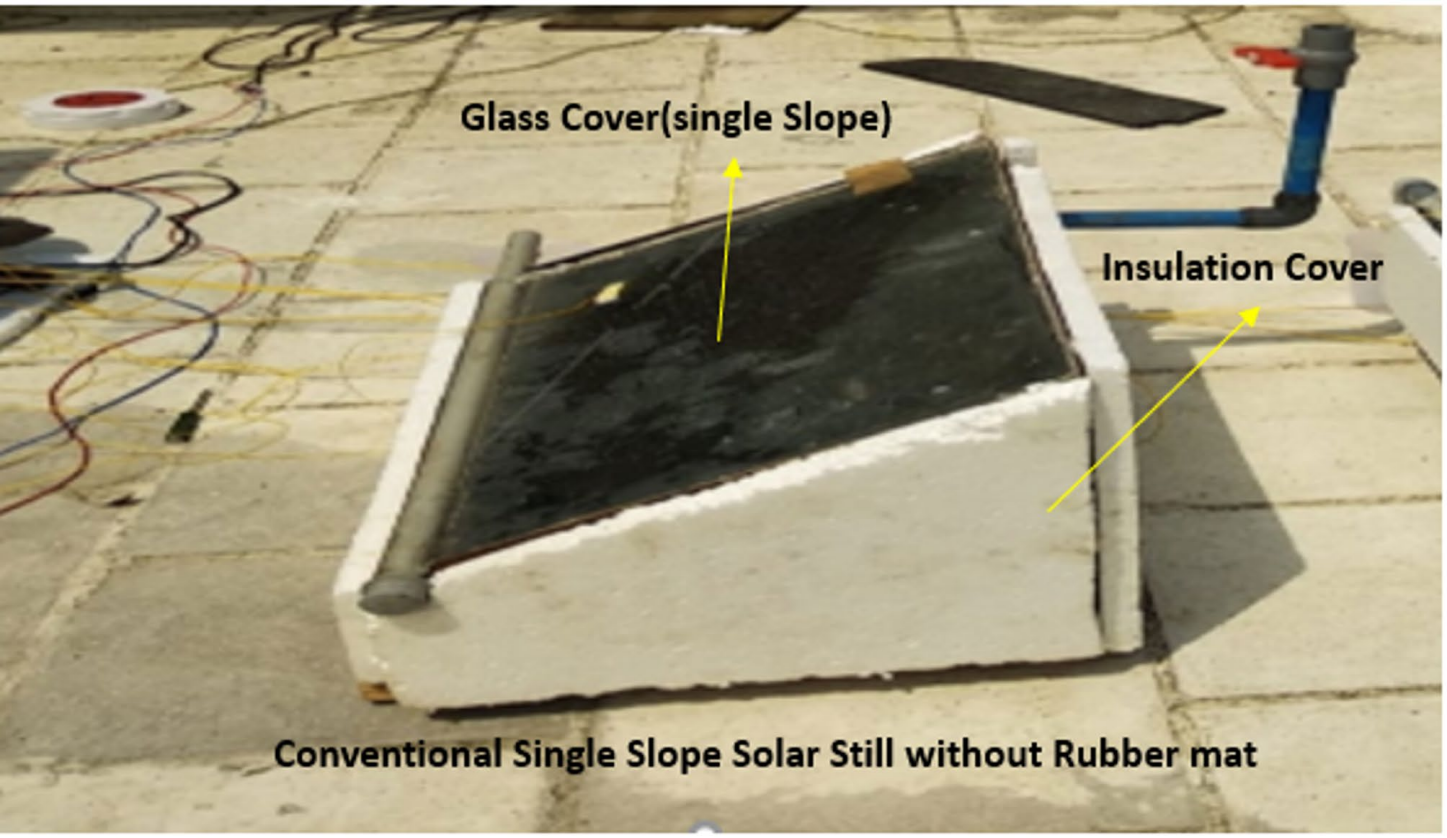
Conventional SS.

**Fig. 2:**
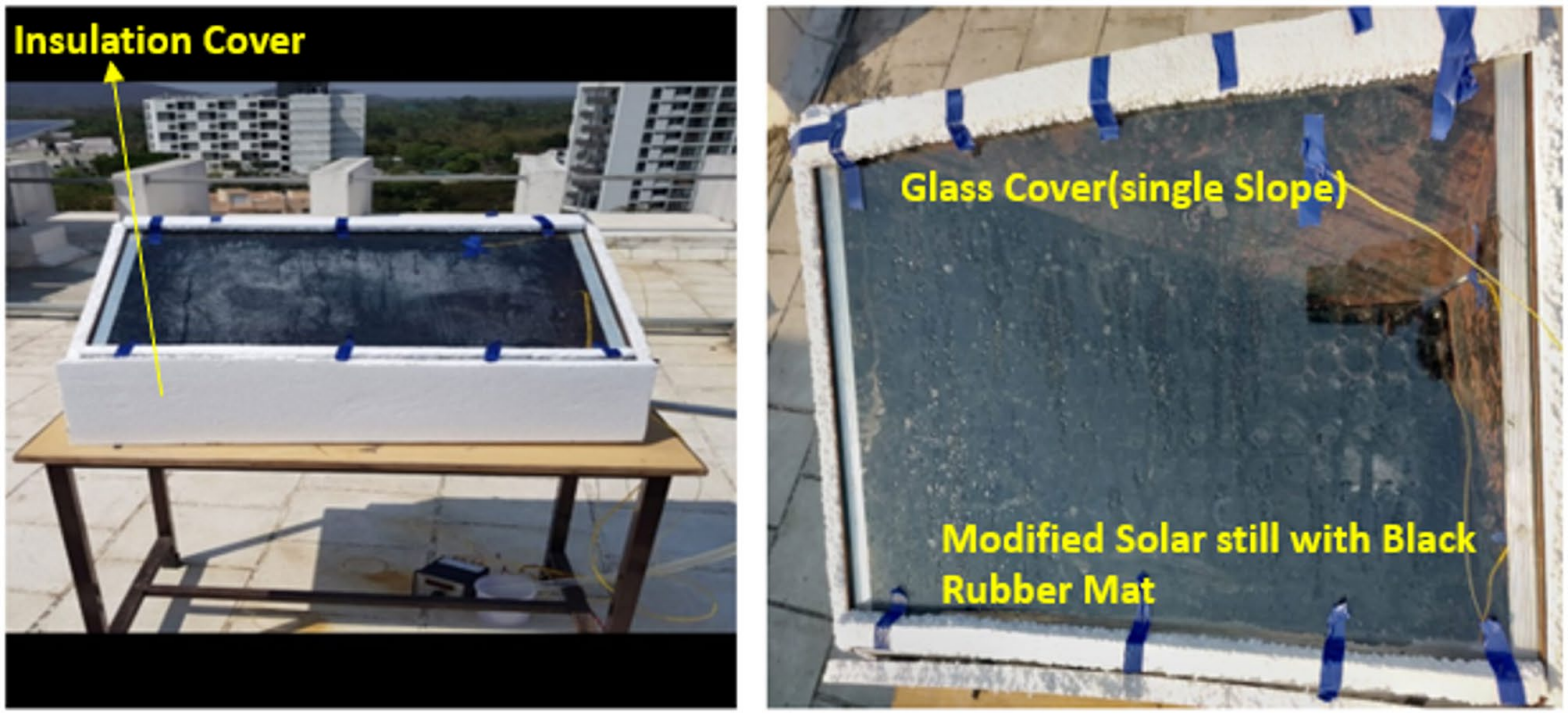
Conventional SS with black rubber mat.

Two solar stills (SS) were constructed using 2 mm-thick mild steel, shaped and welded with arc welding techniques. Each SS featured a 4 mm-thick transparent glass panel inclined at a 32-degree angle to facilitate water collection via an aluminium sheet at the glass’s lower edge. Both solar stills had a base and a floor area of 0.25 m². CSS is illustrated in Figs. 1, and 2 shows the conventional SS with a black rubber mat. Research was conducted at the School of Law, B.S.A.R. Crescent Institute of Science and Technology, Vandalur (latitude of 12.8924 and longitude of 80.08079), Chennai, India. Properties of the black rubber hole mat are mentioned in the Table 1. One of the SS units was fitted with a black rubber mat at the bottom, while both the solar still systems were thermally protected with thermocouples to mitigate heat loss. The experiment, conducted on September 10, 2019, collected data from 8:00 am to 6:00 pm to examine solar radiation’s thermal behaviour, yield, and intensity. Measurements were recorded hourly for water temperature (Tw), glass temperature (Tg), thermal efficiency (TE), and exergy efficiency (EE), with water depth kept constant across both systems, including the one with the black rubber mat. The black rubber mat was an effective energy storage material, with its thermal conductivity (K = 1.7 W/mK) allowing for efficient heat transfer. The perforations present in the mat effectively contained water. They divided the total water volume, facilitating accelerated evaporation of the minor water constituents, subsequently enhancing the TSS system’s overall efficiency. This novel configuration exhibited improved thermal performance compared to conventional systems devoid of the mat.

**Table 1:** Properties of black rubber hole mat.

Appearance	Dense
Colour	Black
Thermal conductivity	1.7 W/mK
Specific heat capacity	1.3 J/kgK
Young’s modulus of elasticity	0.05 Gpa
Water absorption	0%
Density	1100 kg/m^3^

## Results and discussion

### SS and SS with a rubber mat underwent hourly variations in several parameters (09-10-2019)

**Fig. 3:**
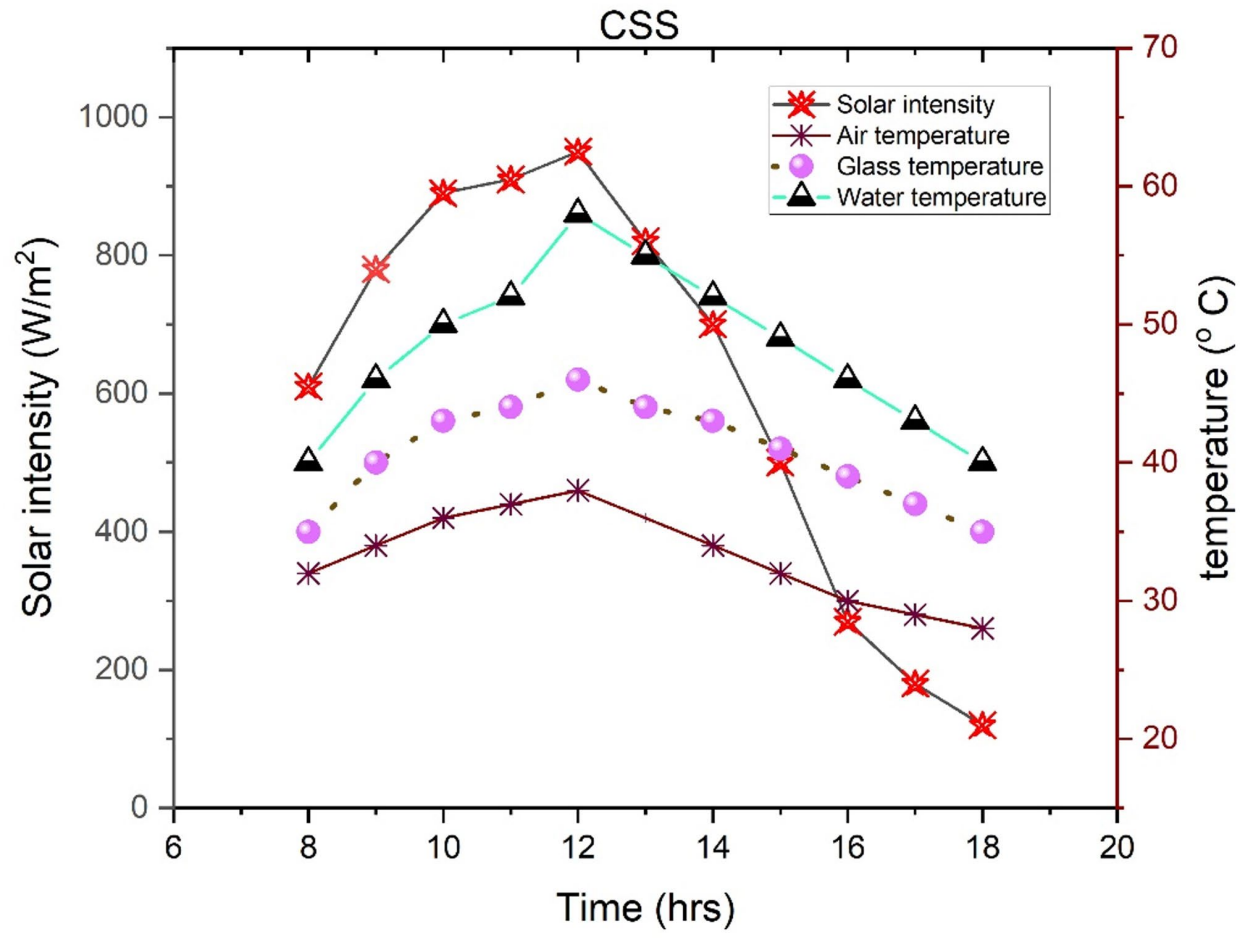
Fluctuations of different parameters in the SS observed on an hourly basis (09-10-2019).

**Fig. 4:**
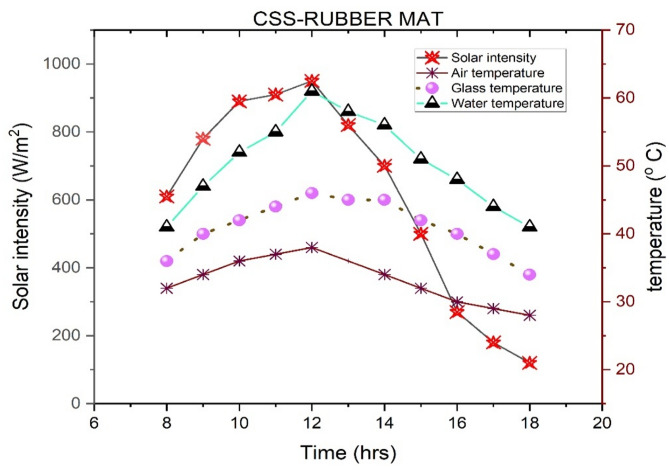
Variations of different parameters in the SS with a rubber mat were observed on an hourly basis (09-10-2019).

Figures 3 and 4 depict the diurnal variations of parameters for the solar still (SS) and the SS integrated with a rubber mat on September 10, 2019. The apex of solar radiation was recorded at noon, attaining a value of 950 W/m², with a daily mean of 611.818 W/m². By 18:00 h, the solar radiation diminished to 120 W/m². The ambient temperature (Ta) reached its zenith at 38 °C at noon, whereas the average Ta was calculated to be 33.273 °C. In the case of the SS devoid of the rubber mat, the mean glass temperature (Tg) and water temperature (Tw) were measured at 40.64 °C and 48.27 °C, respectively, with Tw attaining a maximum of 58 °C at noon. In contrast, the SS with the rubber mat demonstrated average Tg and Tw values of 41 °C and 50.36 °C, respectively. In this setup, it rose from 41 °C at 8:00 am to a maximum of 61 °C at noon, then gradually declined to 41 °C by 6:00 pm. Tg increased from 36 °C at 8:00 am to a high of 46 °C at noon, followed by a drop to 34 °C by the day’s end. At noon, the maximum Tg and Tw for the SS with the rubber mat were 46 °C and 61 °C, respectively.

These findings highlight the substantial influence of temperature variations and solar radiation intensity on the overall efficiency of both solar still configurations. Additionally, factors such as ambient humidity, wind speed, and duration of sunlight exposure can further affect their thermal performance and freshwater yield. The variations align with Vembu et al.^53^, demonstrating that higher temperatures generally enhance evaporation rates, increasing water yield. Conversely, lower temperatures reduce evaporation, decreasing efficiency. Additionally, solar radiation levels play a crucial role by providing energy for evaporation; more significant solar radiation boosts heat transfer and evaporation. In contrast, reduced solar radiation limits energy input and lowers efficiency, as Mahian et al.^54^ highlighted. The improved performance of the SS equipped with a rubber mat compared to the SS can be mainly attributed to the improved thermal properties of the rubber mat. The darker color and higher solar absorptivity of the rubber absorb more incident solar radiation, thereby increasing the heat transfer to the water. Furthermore, its low thermal conductivity acts as an insulating layer, minimizing heat losses from the basin structure and retaining more energy in the basin water. As observed in the experimental data, leading to an enhancement of the temperature of the liquid, which significantly increases the evaporation rate, since evaporation increases exponentially with increasing temperature. The rubber mat also promotes a more uniform and continuous heat release, allowing continuous heating of the water throughout the day. Overall, the rubber mat improves thermal efficiency, thereby increasing distillate yields in the SS equipped with a rubber mat.

### Hourly change of EHTC AND water yield of the CSS and SS with rubber mat on 09-10-2019

**Fig. 5:**
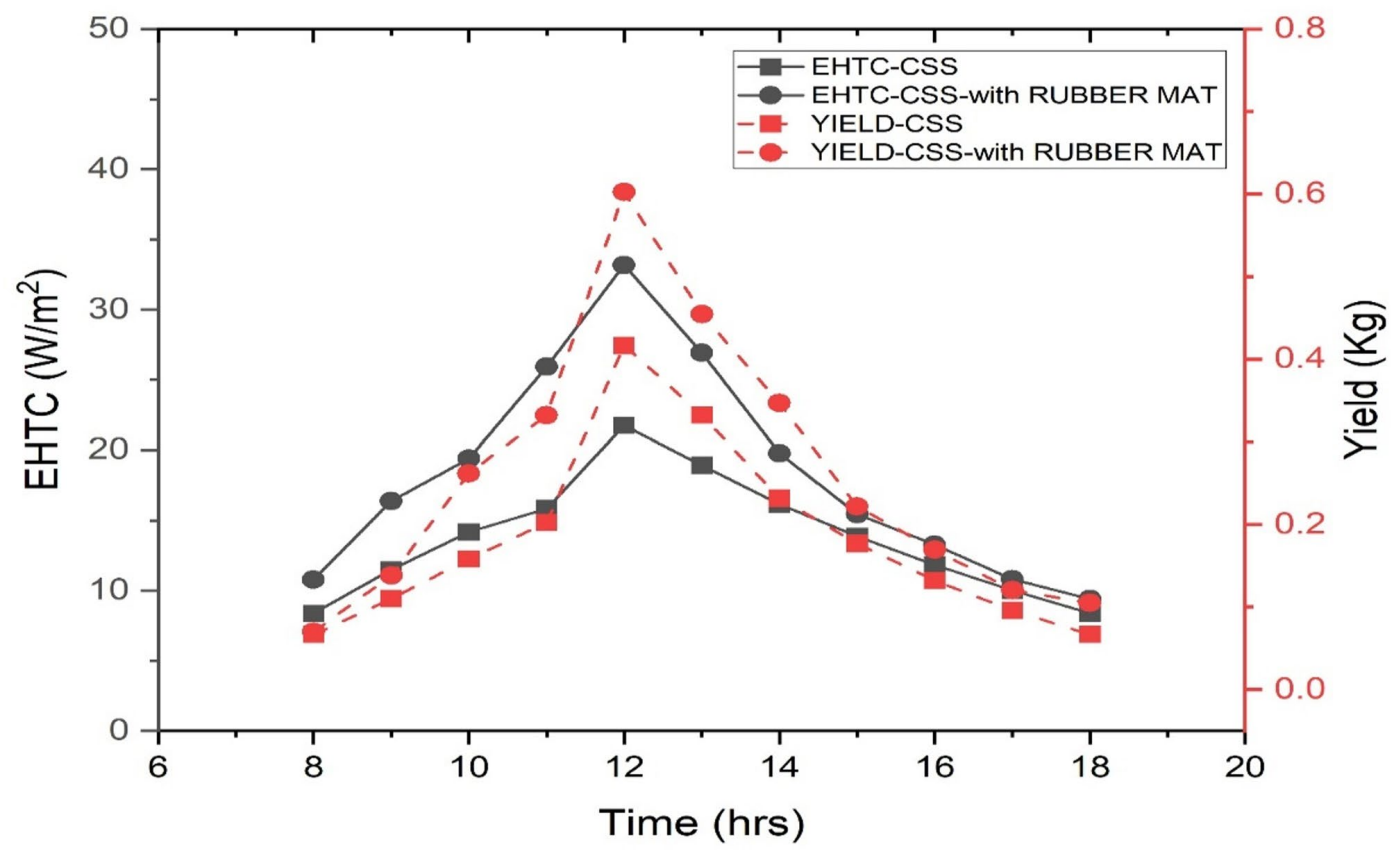
Freshwater production & EHTC of the SS & SS with rubber mat on 09-10-2019 at hourly basis.

This section presents the results of the hourly changes in the EHTC and productivity of freshwater generation for the CSS and the SS incorporated with a rubber mat on September 10, 2019. As shown in Fig. 5, the EHTC for both setups gradually increased until noon, with the CSS reaching the highest of 21.75 W/m²K at noon. In comparison, the SS with the rubber mat achieved a higher peak EHTC of 33.17 W/m² K. The average EHTC values for the day were 13.71 W/m² K for the CSS and 18.29 W/m² K for the SS with the rubber mat. By 6:00 pm, the minimum EHTC values were 8.38 W/m² K for the CSS system and 9.40 W/m² K for the SS with the rubber mat.

The overall freshwater generated from the CSS system was 1.99 kg. In contrast, the SS system incorporated with the black rubber mat recorded the highest water yield hourly, 0.60 kg at noon, contributing to a total daily yield of 2.82 kg. These results indicate that the SS with the rubber mat outperformed the conventional SS in EHTC and water productivity.

The increased EHTC values for the SS with the rubber mat suggest that the mat provided improved insulation and heat transfer efficiency, consistent with the findings of Chauhan et al.^55^. The rubber mat’s higher thermal conductivity likely contributed to this enhanced performance, allowing for more efficient evaporation and increased water yield. The average EHTC values of 13.71 W/m² K for the conventional SS system and 18.29 W/m² K for the SS with the rubber mat reflect the overall thermal performance of both systems during the observation period. Betz et al.^56^ noted that lower EHTC values indicate reduced heat transfer efficiency, while higher values correspond to better performance.

The improved performance of the SS with the rubber mat is reflected in a higher EHTC and distillate yield compared to the conventional SS. This can be scientifically attributed to the improved heat absorption and insulation properties of the rubber mat. The darker color of the rubber mat surface absorbs more solar energy, which can capture more solar energy, thereby increasing the temperature of the still basin and accelerating the evaporation rate of water. In addition, its low thermal conductivity minimizes heat loss from the still basin structure, ensuring that more heat is available for evaporation. This improvement effectively enhances the thermal performance of the SS by maximizing energy utilization and maintaining higher water temperatures throughout operation.

### Variation of EE and TE of CSS and SS system using a rubber mat on 09-10-2019

**Fig. 6:**
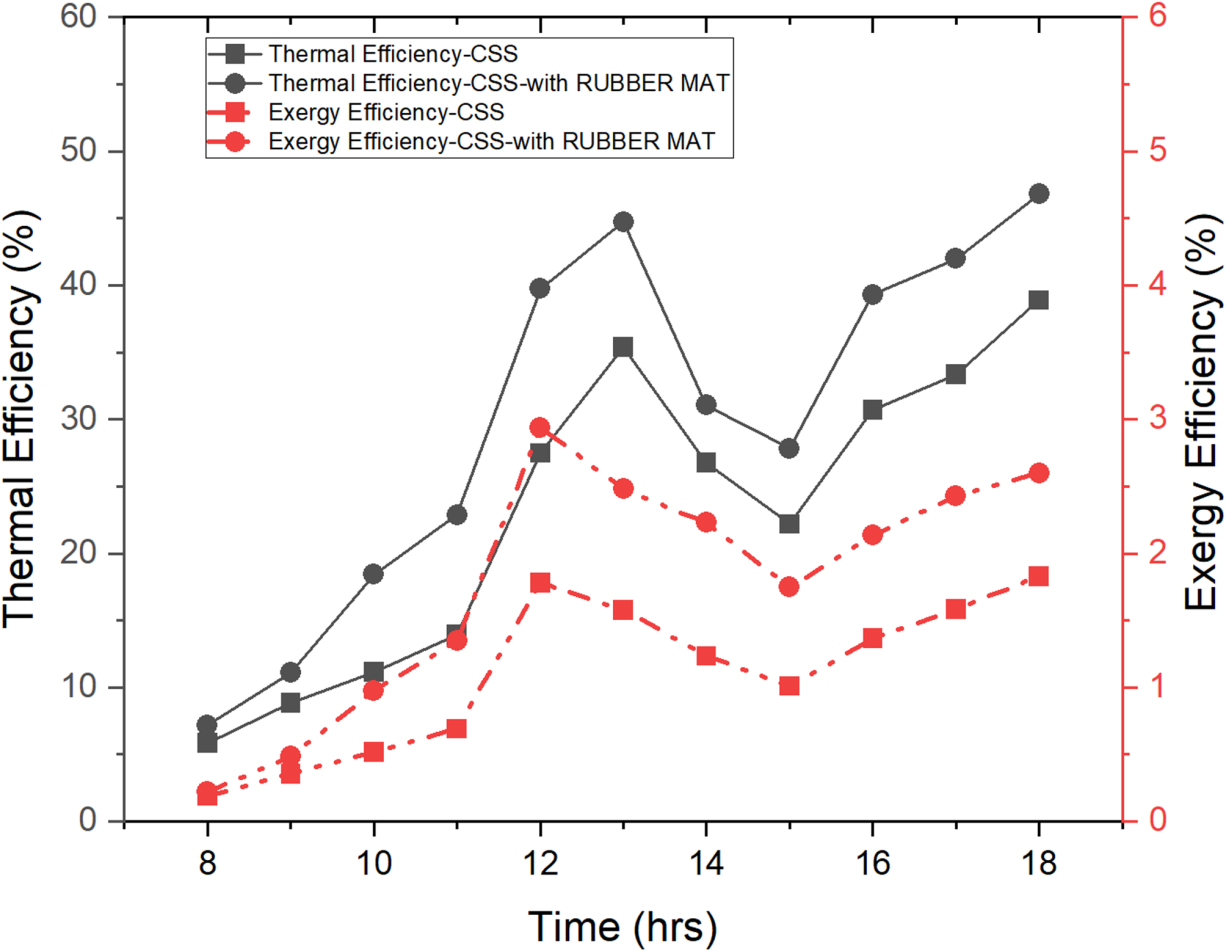
Variations of thermal & exergy efficiency of CSS & SS using a rubber mat on 09-10-2019.

This section examines the diurnal fluctuations of first-law and second-law efficiency for both the solar still (SS) and the SS utilizing a rubber mat, as documented on September 10, 2019. The data presented in Fig. 6 elucidates significant disparities between the two systems regarding their efficiency metrics. At 6:00 pm, the SS attained its peak TE of 34%, with a mean TE of 21.412% throughout the day. TE quantifies the ratio of useful energy output (freshwater production) to the input energy from the intensity of solar radiation that it still receives, whereby elevated values signify a more efficient utilization of energy resources. In contrast, the SS with the rubber mat, acting as a Low-Cost Energy Storage Material (LCESM), reached a peak value of TE at 54.80% at 6 pm, despite the intensity of solar energy being 120 W/m². Despite low solar energy, the high value of TE at 6 pm demonstrates the rubber mat’s capacity to improve the system efficiency by storing and utilizing energy effectively, even as solar radiation decreased. The average TE for the SS with the rubber mat was 29.915%, reflecting a significant improvement over the conventional SS.

The exergy efficiency (EE), which evaluates the system’s ability to convert available energy into practical work (water production), is also improved with the rubber mat. The SS without the rubber mat had a maximum EE of 1.78% and an average EE of 1.0659%. The SS attained a higher peak EE of 2.94% with the rubber mat, signifying enhanced energy conversion efficiency. The increased EE can be attributed to the rubber mat’s ability to minimize energy losses and optimize exergy performance, aligning with findings from Sivakumar^57^.

The significantly improved energy and exergy performance of the SS with rubber mat compared to the conventional SS can be scientifically attributed to the improved properties of the rubber as energy storage substance used in the basin. The rubber mat, with its high solar absorptivity and low thermal conductivity, absorbs more solar radiation and retains heat for a longer time, minimizing loss to the environment. This results in an increase in water temperature, which increases the energy available for evaporation. As a result, indicating that the absorbed solar energy can be more efficiently utilized for useful work. The increase in exergy also reflects the improved thermodynamic quality of energy conversion in the improved system (SS with rubber mat). This demonstrates that the integrated rubber mat can improve productivity in solar desalination systems.

Table 1 presents a comprehensive summary of potable water productivity from various previous research studies, highlighting the utilization of different ESMs based on their availability and effectiveness in other countries. For instance, Kabeel et al.^58^ conducted a survey in Egypt utilizing graphite material as SHESM, achieving an output of about 7.74 L/m². Attia^59^ employed small balls of aluminium material in the solar still, yielding 6.25 kg of water in Algeria. Similarly, Suraparaju et al.^60^ reported a yield of 2950 mL/m²day using ball marbles in India.

In contrast, the present study, which incorporated a black rubber mat as SHESM in the solar still, achieved a yield of 2.82 kg, while the solar still without the rubber mat yielded 1.99 kg. This suggests that adding the rubber mat enhanced the water yield, although it was lower than the yields reported in Egypt and Algeria. The higher yields reported in countries like Egypt and Algeria can be attributed to the more favourable climatic conditions, such as higher solar radiation and more consistent sunshine, which may have contributed to the increased efficacy.

However, some studies conducted in India, such as those by Modi^27^, Mevada^44^, S.Noman^61^ and Kadhim Hussein^62^, reported lower yields than the present research. These variations in productivity highlight the influence of local environmental conditions, including solar radiation, temperature, and the specific design and materials used in each study (Table 2).

**Table 2:** Comparison of present research with identical studies.

Reference	Country	Material used	Yield
Kabeel (2018)^58^	Egypt	Graphite	7.74 L/day m^2^
Modi (2020)^63^	India	Black granite	2253.6 ml/m^2^
Suraparaju (2021)^60^	India	Ball marbles	2950 mL/m^2^
Kumaravel (2023)^64^		Blue metal stones, pebble stones,	
Sambare (2023)^65^	India	Jute cloth, iron pieces, and wire mesh	4.03,4.74 & 5.23 L/m^2^
Mevada^44^	India	Black colour glass ball (BCGB), Black granite (BG), and White marble stone (WMS)	2.50 L/ m^2^
Attia (2020)^59^	Algeria	Aluminium balls	6.25 kg
Noman^66^	India	Custard apple seeds	3.73 kg/m^2^
Noman^61^	India	Pistachio shell powder	2.7 kg/m^2^
Hussein (2023)^62^	India	Salt balls 1.5 cm water depth for (case 1) salt balls and 2 cm water depth for (case 2)	1934 g/ m^2^ 1655 g/ m^2^
Present work	India	Black rubber mat	2.82 kg

### Economic analysis for CSS and SS using a rubber mat

Economic analysis is performed for all the desalination system to estimate the cost per one liter (CPL) of distillate produced and the payback period (PBP) of the desalination system. The principal investment is based on the price of each part of the desalination system. The following assumptions were made for economic analysis^67,68^.

The economic analysis of solar still with CPL and PBP is given by the following equations.

- The Annual First Cost (AFC) is given by:
$${\text{AFC}}\,=\,{\text{CRF}} \times {\text{PI}} - \,{\text{1}}$$
  Where CRF is the Capital Recovery Factor and PI denotes the Principal Investment, and that
  $$\:\text{C}\text{R}\text{F}=\frac{\text{y}{\left(1+\text{y}\right)}^{\text{x}}}{{\:\:\left(1+\text{y}\right)}^{\text{x}}-1}$$.
  Where: y denotes interest rate.
  X indicates the lifetime of the desalination system.
- The Annual Salvage Cost (ASC) is given by,
  $${\text{ASC}}\,=\,{\text{SFF}} \times {\text{SV}} - \,{\text{2}}$$
  Where SFF is the sinking fund factor and SV is the salvage value
  $$\:\text{S}\text{F}\text{F}=\frac{\text{y}}{{\:\:\left(1+\text{y}\right)}^{\text{x}}-1}$$.
  Where: y denotes interest rate.
  X indicates the lifetime of the desalination system.
  $${\text{SV}}\,=\,{\text{2}}0\% \times {\text{P}}$$
- The Annual Maintenance Cost (AMC) per year is expected to be 15% of AFC and hence,
  $${\text{AMC}}\,=\,{\text{15}}\% \times {\text{AFC}} - \,{\text{3}}$$
- The Total Annual Cost (TAC) is given by:
  $${\text{TAC}}\,=\,{\text{AFC}}\,+\,{\text{AMC}}--{\text{ASC}} - \,{\text{4}}$$
- The Total Cost per Liter (CPL) is given by:
  $$\:\text{C}\text{P}\text{L}=\frac{\text{T}\text{A}\text{C}}{\text{A}\text{A}\text{Y}}-5$$
  Where Average Annual Yield (AAY) is given by.
  $$\:\text{A}\text{A}\text{Y}=\left\{\text{N}\text{u}\text{m}\text{b}\text{e}\text{r}\:\text{o}\text{f}\:\text{s}\text{u}\text{n}\text{n}\text{y}\:\text{d}\text{a}\text{y}\text{s}\times\:|\text{A}\text{v}\text{e}\text{r}\text{a}\text{g}\text{e}\:\text{y}\text{i}\text{e}\text{l}\text{d}\:\text{o}\text{n}\:\text{a}\:\text{d}\text{a}\text{y}\right\}$$
- The Pay Back Period.
  $$\:\:\text{P}\text{B}\text{P}=\frac{\text{P}\text{r}\text{i}\text{n}\text{c}\text{i}\text{p}\text{a}\text{l}\:\text{I}\text{n}\text{v}\text{e}\text{s}\text{t}\text{m}\text{e}\text{n}\text{t}\left(\text{P}\text{I}\right)}{\text{N}\text{e}\text{t}\:\text{E}\text{a}\text{r}\text{n}\text{i}\text{n}\text{g}\:\left(\text{N}\text{E}\right)}-6$$
  Where Net earnings (NE) is given by$\:\text{A}\text{A}\text{Y}=\left\{\text{M}\text{a}\text{r}\text{k}\text{e}\text{t}\:\text{p}\text{r}\text{i}\text{c}\text{e}\:\text{o}\text{f}\:\text{w}\text{a}\text{t}\text{e}\text{r}\times\:|\text{D}\text{a}\text{i}\text{l}\text{y}\:\text{p}\text{r}\text{o}\text{d}\text{u}\text{c}\text{t}\text{i}\text{v}\text{i}\text{t}\text{y}\right\}$.
- The selling price of distillate water for the calculations is taken as Rs 20 per litre.
- The life span of the solar still is considered to 10 years.
- The interest rate is also 12%.
- 15% annual maintenance price is considered.

Table 3 listed the cost of components for the CSS with and without black rubber hole mat, And Table 4 listed the parameter and outcomes of economic analysis on CSS with and without black rubber hole mat. The economic analysis clearly shows that the integration of black rubber mats into a SS improves the cost-effectiveness and overall system performance. Although the initial capital investment (PI) and annual fixed cost (AFC) of the SS using a rubber mat is slightly higher due to the added materials, these costs are offset by significantly higher annual production (AAy = 761.4 L/m² vs. 537.3 L/m²) and net benefits (NE = ₹56.4 vs. ₹39.8). The increased productivity results in a significantly shorter payback period (PBP), with the SS using a rubber mat in just 70.92 days, compared to 98.72 days for the conventional SS. The higher residual value (SV), annual maintenance cost (AMC) and annualized residual value (ASC) remain proportionally consistent, indicating that the inclusion of the rubber blanket improves performance without introducing disproportionate operational or disposal costs. Overall, the use of black rubber mat resulted in better economic returns, shorter payback periods and higher water yields, making it an economically viable and technically superior improvement to solar distillation systems.

**Table 3:** Cost of components for the CSS with and without black rubber hole mat.

Principal investments	CSS	CSS with B *R* H M
Mild steel plate	2000	2000
Transparent glass cover	300	300
P V C pipe	100	100
Feed water tank	300	300
Insulation material	50	50
Measuring Jar	50	50
Flexible hole	50	50
Black rubber hole mat	000	150
Installation and testing	1000	1000
Total cost in rupees	3850	4000

**Table 4:** Parameter and outcomes of economic analysis on CSS with and without black rubber hole mat.

Parameters	CSS	CSS with B *R* H M
Life of the system (x)	10	10
Interest rate (y)	0.12	0.12
Principal Interest (PI)	3850	4000
C R F	0.1769	0.1769
A F C	681.065	707.6
S F F	0.0569	0.0569
S V	770	800
A M C	102.159	106.14
A S C	43.81	45.52
T A C	739.414	768.22
C P L	1.3769	1.0089
A A Y	537.3	761.4
N E	39.8	56.4
P B P	98.72 days	70.92 days

## Conclusion

Two SS were manufactured and experimented with. Their performance conditions were compared. One still contains a rubber mat at the basin, and the other does without. Both stills were studied from 8:00 am to 6:00 pm, and the following conclusions are summarized:

- The yield of SS was 1.99 Kg. At noon, the maximum productivity for SS with a rubber mat was 0.60 kg. The total yield of SS with rubber mat was 2.82 kg.
- The maximum TE achieved by SS was 34.90%. At 6:00 pm, the maximum TE attained with a rubber mat was 54.80%.
- The inclusion of the rubber mat at the basin of the SS enhanced productivity and efficiency. Nafey et al.^69^ and Akash et al.^51^ experimented on the black rubber mat to determine the generation of freshwater output. Their experimental water generation was enhanced by 20% and 38%, respectively.
- This study concluded that the black rubber mat used is a good alternative energy storage material. The mat has several holes that can be used for trapping water, thus separating the whole mass of water in an SS into several separate water masses. In the separate water masses, evaporation occurs faster and increases efficiency compared to other studies by some researchers.
- Comparing the SS to the SS with a rubber mat reveals that including a rubber mat at the basin enhances both productivity and efficiency. Therefore, it is recommended to incorporate a rubber mat in the design of solar stills to augment their performance.

### Recommendation

Based on the given findings and conclusions, the following recommendations are made:

- Explore alternative energy storage materials: The black rubber mat used in the study proved to be a good alternative energy storage material. This indicates that other materials with similar properties can also enhance the efficacy of a desalination process based on solar energy. Further research can focus on exploring and testing different energy storage materials to identify the most suitable options.
- Utilized water-trapping holes in the mat: Holes in the rubber mat allowed for water trapping, creating separate water masses within the SS. This separation resulted in faster evaporation and increased efficiency. Therefore, future designs can consider incorporating similar water-trapping mechanisms or structures to enhance the efficiency of the solar stills.
- Build on previous research: The findings of Nafey et al.^69^ demonstrated increased productivity using a black rubber mat. This research supports their findings and provides additional evidence for the effectiveness of such mats. Future studies can build upon these previous works to further investigate and optimize rubber mats used in solar desalination systems.

## Supplementary Information

Below is the link to the electronic supplementary material.

Supplementary Material 1

## Data Availability

All data are given in the manuscript.

## Abbreviations

SS: Conventional solar still
PSS: Pyramid solar still
TSPSS: Triangle shaped pyramid solar still
DBSS: Double basin solar still
SSDBSS: Single slope double basin solar still
SBDSSS: Single basin double slope solar still
TSS: Tubular solar still
SSSS: Single slope solar still
IASS: Inverted absorber solar still
HTCM: High thermal conductivity material
SS: Solar still
SSBSS: Single slope basin solar still
TPSS: Triangular pyramid solar still
LHTESS: Latent heat energy storage
ISS: Inclined solar still
SBSSPSS: Single basin single slope plastic solar still
EE: Exergy efficiency
SBSS: Spherical basin solar still
T_a_: Air temperature
T_w_: Water temperature
PCM: Phase change material
TE: Thermal efficiency
SHSM: Sensible heat storage material
T_g_: Glass temperature
EHTC: Evaporative heat transfer coefficient
ESM: Energy storage materials
