# Supplementary material for: Performance improvement of solar still by water mass splitting arrangement
Source: Sci Rep. 2025 Aug 22;15:30965. doi: 10.1038/s41598-025-15849-1 (PMC12373784; doi:10.1038/s41598-025-15849-1)
Supplement: Supplementary file 1 — Supplementary Material 1 [file 41598_2025_15849_MOESM1_ESM.doc]

# APPENDIX-1

## Errors from Experimental Work

Experimental errors encompass deviations or inaccuracies in measurements or results attributable to limitations inherent in instruments, human error, environmental influences, or procedural inadequacies. These errors may manifest as systematic (consistent bias) or random (unpredictable variations). Identifying and minimizing such errors is paramount for ensuring the reliability and validity of experimental results. Solar radiation, temperature, and water collection measurements are executed hourly using specialized apparatus. A flask is utilized to quantify the volume of water collected, whereas a K-type thermocouple is employed to gauge temperature. A solar power meter is deployed to evaluate solar radiation levels, displaying temperature readings on a digital thermometer. Table 1 delineates the associated errors for the instruments utilized in these measurements.

**Table 4**: measurement device error

| **Equipment** | **accurateness** | **Spectrum** | **Error** |
| --- | --- | --- | --- |
| Temperature sensors | ± 0.1^o^C | -100 to 600^o^C | ±1.5% |
| Flask (for water collection) | ± 10 ml | 0-1500 ml | ± 1.5% |
| Solar meter | ±10 W/m^2^ | 0-3500 W/m^2^ | ± 3.5 % |

## Uncertainty analysis

Any potential errors in the instruments used may contribute to the overall uncertainty observed in the study. Table 4 presents the level of uncertainty associated with each experimental instrument, presented in a formatted manner.


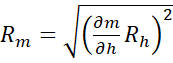
 (9)

The calculation of daily efficacy for the SS and SS with a rubber mat is subject to uncertainty, which is influenced by solar intensity and yield. This information is presented in a formatted manner.


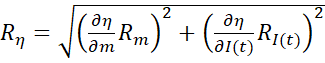
 (10)

The anticipated variabilities associated with assessing diverse parameters, such as the intensity of solar radiation, were quantified utilizing a solar meter, yielding a value of 3.5%. Similarly, the uncertainty about the temperatures of all examined parameters—including water, air, and glass—was determined to be 1.5%. Furthermore, the uncertainty associated with freshwater productivity derived from the experimental analysis was 1.5%. In contrast, the uncertainty regarding the first law efficiency of the solar still was articulated as 2.1%, facilitated by the application of equation (10).

## APPENDIX-2

The calculation of EHTC between salt water and the acrylic glass is determined using the equation^70,71,72^

“
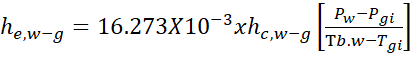


The convective heat transfer coefficient from the briny water to the glass cover is calculated by,


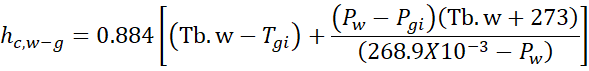


Partial vapour pressure at the T_b.w_is calculated by,


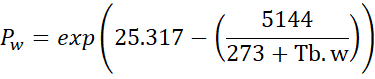


Partial vapour pressure at the glass surface is calculated by,


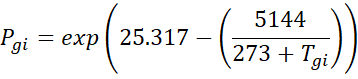


The thermal efficiency of the SS and SS with a rubber mat is estimated as,


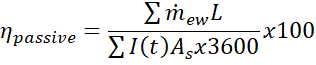


The exergy effectiveness of the SS and SS with a rubber mat is given by,

$$\eta_{overall,exe}=\frac{\sum Ex_{output}}{\sum Ex_{input}}$$

The hourly exergy output is calculated by,

$$Ex_{output}=\frac{m_{ew}L_{fg}}{3600}\times\left[1-\frac{T_{a}}{T_{w}}\right]$$

The hourly exergy input is calculated by,

$Ex_{input}=A_{w}I^{'}(t)\times\left[1-\frac{4}{3}\left(\frac{T_{a}}{T_{s}}\right)+\frac{1}{3}\left(\frac{T_{a}}{T_{s}}\right)^{4}\right]$”
